# Biodegradable Mg–Zn–Ca-Based Metallic Glasses

**DOI:** 10.3390/ma15062172

**Published:** 2022-03-15

**Authors:** Chao Jin, Zhiyuan Liu, Wei Yu, Chunling Qin, Hui Yu, Zhifeng Wang

**Affiliations:** 1 Key Laboratory for New Type of Functional Materials in Hebei Province, School of Materials Science and Engineering, Hebei University of Technology, Tianjin 300401, China; jinchaohebutmail@163.com (C.J.); clqin@hebut.edu.cn (C.Q.); yuhuidavid@gmail.com (H.Y.); 2 Research Institute of Foundry, Hebei University of Technology, Tianjin 300401, China; 3 HNC Key Laboratory of Hormones and Development, Tianjin Key Laboratory of Metabolic Diseases, Chu Hisen-I Memorial Hospital, Tianjin Medical University, Tianjin 300134, China; 4 Tianjin Institute of Endocrinology, Tianjin Medical University, Tianjin 300134, China; 5 School of Materials Science and Engineering, Hefei University of Technology, Hefei 230009, China; yuwei52213@163.com; 6 Hefei Nova Advanced Materials Company Ltd., Hefei 230000, China

**Keywords:** metallic glass, Mg–Zn–Ca, biodegradable, implant

## Abstract

Biodegradable Mg–Zn–Ca-based metallic glasses (MGs) present improved strength and superior corrosion resistance, compared to crystalline Mg. In particular, in vivo and in vitro attempts reveal that biodegradable Mg–Zn–Ca-based MGs possess excellent biocompatibility, suggesting that they are ideal candidates for temporary implant materials. However, the limited size and severe brittleness prevent their widespread commercialization. In this review, we firstly summarize the microstructure characteristic and mechanical properties of Mg–Zn–Ca-based MGs. Then, we provide a comprehensive and systematic understanding of the recent progress of the biocorrosion and biocompatibility of Mg–Zn–Ca-based MGs. Last, but not least, the outlook towards the fabrication routes, composition design, structure design, and reinforcement approaches of Mg–Zn–Ca-based MGs are briefly proposed.

## 1. Introduction

Recently, research enthusiasm is mushrooming in Mg-based alloys as biodegradable implants for biomedical application [1,2,3,4,5] for their multifaceted advantages. Firstly, Mg-based alloys exhibit high strength and comparable Young’s modulus to that of cortical bone. Secondly, their natural biodegradability makes it possible to eliminate the second surgery for implant removal, after the completion of tissue healing [6]. Thirdly, magnesium is a fundamental ingredient in the human body, and its recommended daily intake for adults is 240–420 mg day^−1^ [7], implying the good biocompatibility of Mg-based alloys. Last, but not least, Mg is involved in many biological functions, such as bone growth and the stabilization of genomic structures [8,9]. Due to these excellent properties, Mg-based alloys has been used or shown enormous potential in cardiovascular stents, MAGNEZIX screw, microclip for laryngeal microsurgery, biodegradable orthopedic implants, and wound-closing devices applications [10]. Nevertheless, the etching speed of Mg-based alloys is rapid, resulting in the generation of a big volume of hydrogen gas at the implant site and increase in local pH value [11]. Alloying is a mainstream method to solve this problem. It has been demonstrated that the incorporation of alloying elements, such as Ca, Zn, Al, rare earth, and Mn, can enhance the erosion resistance of Mg alloys [12]. Owing to the limited solid solubility on Mg, excess addition of alloying elements will produce many precipitates, which can generate micro-galvanic couples with ambient primary Mg and expedite etching kinetics [13].

Metallic glasses (MGs) are chemically homogeneous systems, formed by suppressing the nucleation and growth of crystalline phase in some alloy melts [14]. This makes MGs have neither a regular periodic arrangement of crystal atoms nor grains or grain boundaries, showing a long-range disordered structure. The glass-forming ability (GFA), reflected by the critical diameter (d_c_, maximum diameter, or size of a sample capable of forming MGs), implies the difficulty level it can be fabricated in amorphous form, through rapidly solidifying the melt [15,16]. When cast into a copper mold (a common method for producing MGs), a high GFA suggests a resultant high d_c_ [15]. Bulk metallic glasses (BMGs) can form at very low critical cooling rates (<100 K/s), when compared to that of early MGs (10^5^–10^6^ K/s) and, thus, possess higher possible d_c_ [17,18]. The term “MGs”, used in this review, refers to materials that include BMGs and traditional MGs. The advantages of MGs are their superior specific strength and hardness, high resistance to corrosion and wear, polymer-like formability, and excellent magnetic properties [19,20], due to which they have received widespread research enthusiasm. More importantly, MGs possess relatively flexible composition spaces, in which the contents of alloying elements can be far beyond the solubility [16].

Mg-based MGs, such as Mg–Zn–Ca [21], Mg–Cu–Gd [22], and Mg–Cu–Y–Nd–Ag MGs [23], exhibit significantly enhanced strength over crystalline Mg alloys [15]. In fact, the compression fracture stress of Mg-based MGs can even exceed 1000 MPa [22]. Additionally, Mg-based MGs have remarkably improved corrosion resistance, compared to crystalline Mg alloys [24]. On the one hand, the absence of second phase and microstructural defects avoided a galvanic effect. On the other hand, the corrosion resistance of Mg alloys depends largely on alloying elements in solid solution. With no solid solubility limits, the corrosion resistance of Mg-based MGs can be greatly enhanced [15]. However, the biocompatibility factor is one of the keys for Mg-based MGs in biomedical applications. Most of the Mg-based MGs contain significant amounts of copper, nickel, and/or gadolinium and, thus, are unsuitable for application in bioresorbable implants. Among Mg-based MGs, Mg–Zn–Ca MGs and Mg–Cu–Y–Zn MGs have been demonstrated to exhibit the potential for biomedical applications [24,25]. Nonetheless, only the Mg–Zn–Ca MGs have received much attention in the research community because they have been confirmed to possess high biocompatibility in cytotoxicity tests and histopathology analyses [25,26].

Mg–Zn–Ca MGs combine the good properties of Mg-based alloys and MGs. They possess superior strength, Young’s modulus suitable for orthopedic implants, and good corrosion resistance, based on full biodegradability and good biocompatibility [21,25]. In 2009, Zebrg et al. [25] characterized the biodegradable Mg–Zn–Ca glasses as ideal candidates for biomedical application. This pioneer report sparked interest in Mg–Zn–Ca MGs, especially in the field of biodegradable orthopedic implants. Nonetheless, there are critical limitations that prevent their widespread commercialization. Most Mg–Zn–Ca MGs were produced by the induction-melting/copper mold injection method, with a d_c_ of less than 5 mm [27]. In addition, Mg–Zn–Ca MGs exhibited plastic strain of almost zero [28], which is harmful during processing. Therefore, some strategies, including minor alloying addition, Mg–Zn–Ca MG matrix composites (MGMCs), and surface coating, have been used to enhance the performance of Mg–Zn–Ca MGs.

The engineering and biomedical domains are two of the main application fields of MGs. Over the last decades, Zr-, Ti-, Fe-, Co-, Ni-, and Cu-based MGs have been intensively studied and applied for engineering materials, including structural materials (such as sporting goods and precision gears for micromotors), magnetic materials (such as magnetic cores and choke coils), etc. [20,29]. There are numerous comprehensive and systemic reviews on various MGs for engineering materials application [29,30,31,32,33,34,35,36]. There are also some reviews on Fe-, Ti-, Zr-, Mg-, and Ca-based MGs, which were developed for biomedical purposes [15,37,38,39,40,41,42,43]. In particular, biodegradable Mg-based MGs are very promising candidates for temporary biomedical implants, such as cardiovascular stents, bone screws, and plates. After Mg is combined with single or combination of transition metals and rare earth elements, Mg-based MGs possessed excellent properties and attracted attention for various applications [42,44]. Among Mg-based MGs for biomedical application, bio-safe Mg–Zn–Ca MGs are the only alloy systems which have drawn attention by researchers over the world. However, while Mg–Zn–Ca MGs have been discussed in many reviews [43,45,46]; a review focused on biomedical Mg–Zn–Ca MGs, both in depth and completeness, is still lacking.

As biodegradable metals (BDMs), Mg-based BDMs have been the focus of the attention [47]. The recently studied Mg-based BDMs can be classified into crystalline alloys and MGs, from the perspective of atomic structure. Over the past 20 years, a lot of intensive research has been conducted on biodegradable Mg-based crystalline alloys [10,11,12,48,49,50,51,52,53], and they now have reached the stage of clinical trials [4]. However, the research on biodegradable Mg-based MGs is still in its early stage. Dambatta et al. reviewed the reported biodegradable Mg-based MGs and found that the advantage of Mg–Zn–Ca MGs was remarkable, on the basis of biomedical purpose [42]. More importantly, research in the field of biomedical Mg–Zn–Ca MGs has developed rapidly over the last decade with significant progress. As a consequence, there is a need for a comprehensive and systemic review on biodegradable Mg–Zn–Ca MGs in the field of biodegradable metals.

In the current review, the microstructure, mechanical properties, biocorrosion, and biocompatibility of biodegradable Mg–Zn–Ca-based MGs are discussed. Owing to the appropriate properties of Mg–Zn–Ca-based MGs, these discussions have focused on the application potential of biodegradable implants. The review further summarizes the current status of Mg–Zn–Ca-based MGs. Future developments are also discussed at the end, from the perspectives of fabrication routes, composition design, structure design, and reinforcement approaches.

## 2. Microstructure and Mechanical Properties of Mg–Zn–Ca-Based MGs

### 2.1. Microstructure

#### 2.1.1. Mg–Zn–Ca MGs

**Figure 1:**
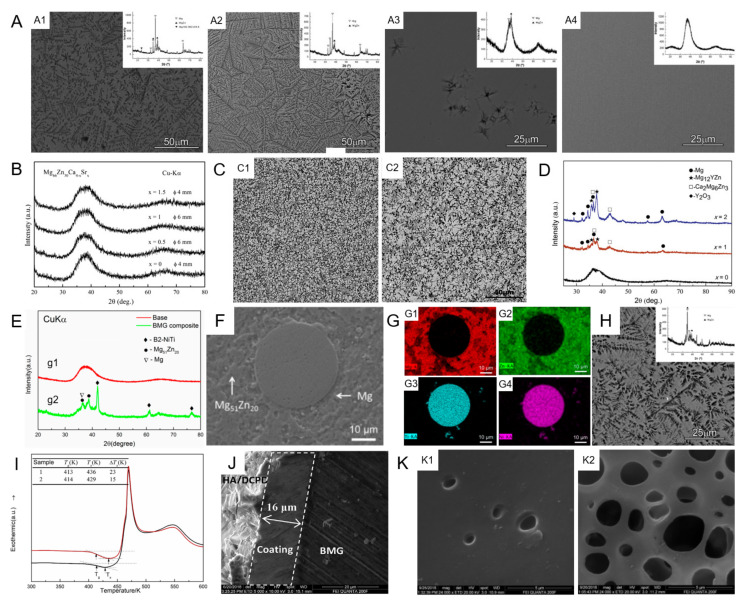
Microstructure of Mg–Zn–Ca-based MGs. (**A**) SEM images of as-cast alloys (diameter: 3 mm) with different Ca ratios: (**A1**) Mg_71_Zn_28_Ca_1_, (**A2**) Mg_70_Zn_28_Ca_2_, (**A3**) Mg_69_Zn_28_Ca_3_, and (**A4**) Mg_68_Zn_28_Ca_4_ alloys. The inset presents XRD patterns for the corresponding alloy, reproduced with permission from [27]. (**B**) XRD patterns of the Mg_66_Zn_30_Ca_4−x_Sr_x_ (x = 0, 0.5, 1, and 1.5 at.%) MGs, reproduced with permission from [58]. (**C**) SEM images of the Mg_69−x_Zn_27_Ca_4_Y_x_ alloys: (**C1**) x = 1, (**C2**) x = 2 at.%, reproduced with permission from [59]. (**D**) XRD patterns of the Mg_69−x_Zn_27_Ca_4_Y_x_ (x = 0, 1, and 2 at.%) alloys [59]. (**E**) XRD patterns of (g1) Mg_67_Zn_29_Ca_4_ MG and (g2) Mg–Zn–Ca MGMC, with 3 vol.% porous NiTi addition. (**F**) SEM images of (g2). (**G**) EDX mapping taken from (H) ((**G1**): Mg; (**G2**): Zn; (**G3**): Ni; and (**G4**): Ti) [60]. (**H**) SEM images of Mg_75_Zn_20_Ca_5_ alloy. The inset is the XRD pattern for the alloy, reproduced with permission from [27]. (**I**) DSC curves of Mg_68_Zn_28_Ca_4_ MG matrix (Sample 1) and HA/ZnO-coated MG (Sample 2), reproduced with permission from [61]. (**J**) SEM image of cross-section HA/ZnO coating on MG surface, reproduced with permission from [61]. (**K**) SEM images of (**K1**) Pure PCL and (**K2**) PCL/2%nHA composite coatings, reproduced with permission from [62].

MGs are produced via rapidly solidifying melts to below their glass-transition temperature, suppressing the nucleation and growth of the crystalline phases [16]. Consequently, unlike the crystal counterparts, which may have precipitates and microstructural defects, such as grain boundaries and dislocations, Mg–Zn–Ca MGs are single-phase and chemically homogenous systems. For instance, there were no second phases and a uniform microstructure in Mg_69_Zn_27_Ca_4_ MG, while there was uneven grain size with an average size of 4.91 ± 0.31 μm in the pure Mg [54]. Additionally, the Mg–Zn–Ca MGs have flexible composition spaces in which they can form a single-phase MG, while their microstructure would remain in amorphous states or crystallize when the size of Mg–Zn–Ca MGs increases. Moreover, the small changes in the chemical composition may beyond the MG composition area, resulting in the precipitations of crystal phases. These behaviors all depend largely on the GFA and strongly affect the microstructure of Mg–Zn–Ca MGs. Owing to the requirement of biomedical applications (such as orthopedic implants), the Mg–Zn–Ca MGs with both a sufficient size and fully amorphous state are of practical significance. Li et al. [27] found that even an increase in 1 at.% Ca caused significant changes in the microstructure of Mg_72−x_Zn_28_Ca_x_ alloys (x = 1~4 at.%), with a diameter of 3 mm. As shown in Figure 1A, the microstructure of Mg_71_Zn_28_Ca_1_ and Mg_70_Zn_28_Ca_2_ alloys (Figure 1(A1,A2)) presents a dendrite shape, while that of the Mg_69_Zn_28_Ca_3_ alloy (Figure 1(A3)) exhibited only a few dendrites. When the Ca content reaches 4% (Figure 1(A4)), there was no contrast in Mg_68_Zn_28_Ca_4_ alloy, revealing that this alloy possessed an amorphous structure. Nowosielski [28] and co-workers concluded that the Ca atomic percentage of 4–5% was the best choice, in order to create the maximum diameter with a completely amorphous state in Mg_69−x_Zn_28_Ca_3+x_, Mg_67−x_Zn_30_Ca_3+x_, and Mg_65−x_Zn_32_Ca_3+x_ (x = 0, 1, 2, and 3 at.%) MGs. By comparing the GFA of Mg_60_Zn_34_Ca_6_ and Mg_73_Zn_23_Ca_4_ MGs, Matias et al. [55] demonstrated that the increase in Zn content would lead to an improvement in the GFA of Mg–Zn–Ca MGs. Although it is a fact that inappropriate compositions can lead to poor GFA, Mg, Zn, and Ca elements can be familiarly mixed at the atomic level and form a single-phase Mg–Zn–Ca MG, due to their fairly wide range of compositions [16]. Besides the GFA, the cooling rate also influence the microstructure of as-cast Mg–Zn–Ca MGs. Alloy rods with a big diameter possess a lower cooling rate at the sample center, owing to the fact that the cooling rate of rod samples declines from the outside to the inside, demonstrating the crystal nucleation and growth at the sample center [56]. To overcome this problem, Song et al. proposed an effective solution to design and synthesize a novel bi-phase core–shell MG composite, including a crystalline Mg core and outer amorphous shell, by two step injection method, for potential application in orthopedic fixation implants [57].

Large critical size is of great practical significance, especially when they are used in orthopedic implants. However, the d_c_ of Mg–Zn–Ca MGs is around several millimeters (Table 1), which is not sufficient for the manufacture of viable orthopedic implants. Therefore, some methods that can effectively increase the size of the material are urgently needed. Among them, minor alloying additions is a common method.

#### 2.1.2. Mg–Zn–Ca MGs with Minor Alloying Additions

The minor addition or microalloying techniques plays important roles in different characteristics, including the formation ability, crystallization behavior, thermal stability, and mechanical property of MGs [64]; hence, these techniques have gathered much attention. As shown in Figure 1B, the XRD patterns of the as-cast Mg_66_Zn_30_Ca_4−x_Sr_x_ (x = 0, 0.5, 1, and 1.5 at.%) alloy rods revealed the amorphous structure of these samples. Furthermore, the d_c_ for Sr0.5 Sr1 alloys were 6 mm, which was an obvious increase, compared to that of 4 mm for Sr0 alloy [58]. The gain of the better GFA may be due to suitable restructuring of an atomic-sized mismatch and more compact local structure, caused by the addition of a proper content of Sr with a bigger atomic size [58]. Additionally, the change in GFA of this Mg–Zn–Ca–Sr MGs was consistent, with the variation in T_rg_ (reduced glass transition temperature, T_rg_ = T_g_/T_l_, T_g_ and T_l_ are the glass transition temperature and liquidus temperature, respectively) and γ (γ = T_x_/(T_g_ + T_l_)); T_x_ is the starting crystallization temperature [58,65]. Furthermore, Wang et al. [66] found that the addition of 0.3~0.5 at.% Sr enhanced the GFA of Mg_65.2_Zn_30_Ca_4_Mn_0.8_ MG, and Sr could restrain the generation of Mn-Zn dendrites. These studies showed that the GFA of Mg–Zn–Ca and Mg–Zn–Ca–Mn MGs could be improved by the addition of trace Sr because the Sr addition made the alloy composition more approximate to the eutectic point. Zai et al. [67] demonstrated that the addition of 1 at.% Ga could increase the GFA of Mg_66_Zn_30_Ca_4_ MG. Taking GFA and binary phase diagrams into consideration, they found that, with the addition of Ga (0~1.25 at.%), the d_c_ of Mg–Zn–Ca–Ga MG declined rapidly to 0.5 mm and then enhanced to 5 mm (peak value) at 1 at.% Ga, which corresponded to the Ca–Ga eutectic composition [67]. However, the addition of minor Mn [68], Cu [69], Y [59], Pd [70], and Ag [71] were not beneficial to the GFA. Figure 1C displayed the backscattered SEM images of the Mg_69-x_Zn_27_Ca_4_Y_x_ (x = 1 and 2 at.%) alloys and showed the disappearance of the single-phase. The XRD patterns (Figure 1D) demonstrated the co-existence of the crystalline and amorphous phases in the Y-doped Mg_68_Zn_27_Ca_4_ alloy [59], which further proved the ruin of GFA. However, it is worth noting that the precipitated phases, shown by the XRD patterns, contained Mg_12_YZn and Ca_2_Mg_6_Zn_3_ phases, both of which were beneficial to the mechanical properties of the matrix [59]. Furthermore, the elements that are introduced to Mg–Zn–Ca MGs must meet biodegradability and biocompatibility criteria before they can be used in biodegradable implants.

#### 2.1.3. Mg–Zn–Ca MGMCs

Monolithic MGs generally fail with the local distortion at ambient temperature for the fast reproduction and transmission of single initial shear bands [72]. To address this restriction, in situ or ex situ methods are commonly used to produce ductile phase reinforced MGs. The ex situ ductile second-phase used to strengthen the Mg–Zn–Ca MG matrixes includes Ti particle [73], porous NiTi particle [60], porous Mo particle [74], and so on. Porous NiTi particles were selected by Guo et al. to introduce into Mg_67_Zn_29_Ca_4_ MG by the ex situ adding process [60]. The XRD patterns and SEM image implied that the ex situ addition of second-phase particles would lead to the reduction of GFA (Figure 1E,F). The EDX element mapping, displayed in Figure 1G, further revealed the microstructure of this Mg–Zn–Ca MGMC. Mg-rich and Mg-poor areas were determined as Mg and Mg_51_Zn_20_ phases, respectively. It also found that Mg, Zn, and Ca from the matrix permeated into the porous NiTi particles, suggesting a good wetting between them [60]. In addition, it is another good choice to form in situ crystalline phases during the solidification process of MGs, which can afford the plastic deformation and impede shear bands at the same time [75]. A typical microstructure of these Mg–Zn–Ca MGMCs was shown in Figure 1H. The dendritic structure was homogeneously distributed in the amorphous matrix and these flower-like crystals were mainly Mg and MgZn intermetallics, which could be indicated from the XRD pattern displayed in the inset [27]. It should be pointed out that some materials formed by the minor addition of elements can also be regarded as composites formed by the in situ addition method, but we do not discuss that in this section.

#### 2.1.4. Surface Coating of Mg–Zn–Ca MGs

Although Mg–Zn–Ca MGs have good corrosion resistance, due to their special amorphous structure, the biodegradation rate still cannot meet the biomedical needs. To make up this shortcoming, Chen et al. prepared a porous and rough silicon-containing coating on the surface of Mg–Zn–Ca MGs by the micro-arc oxidation (MAO) method. The coating, with a thickness of about 12 μm, significantly improved the corrosion resistance of Mg–Zn–Ca MGs [76]. However, this method has some disadvantages. Firstly, the hydroxyapatite (HA) or Si coatings achieved on the MGs by MAO are too thin and uncompact, with numerous visible cracks and holes. Secondly, the HA coating is not easy to adhere to the surface of Mg–Zn–Ca MGs [61]. Consequently, Zhou et al. prepared a dense and thicker nano-HA/ZnO film on the surface of a Mg–Zn–Ca MG by a simple one-step hydrothermal technique in an acid solution [61]. The DSC curves (Figure 1I) implied that the GFA of the MG matrix was reduced because the high temperature would expedite the structural relaxation of MGs. As displayed in Figure 1J, a dense and uniform coating was successfully prepared on the surface of Mg–Zn–Ca MG. The HA/ZnO-coated Mg–Zn–Ca MG presented a modified corrosion resistance, compared to uncoated samples [61]. In addition to effectively reducing the biodegradation rate, the coating suitable for biomedical application should play a role in enhancing the biocompatibility of the MG matrix. It can be seen from Figure 1K that a pure polycaprolactone (PCL) coating on the substrate had a few micropores, with a diameter of less than 1.5 μm (Figure 1(K1)). The PCL/2%nHA coating exhibited higher porosity and larger pore size, due to the addition of nHA (Figure 1(K2)). Although the samples with pure PCL coating showed best electrochemical performance, samples with PCL/2%nHA coating possessed better cytocompatibility, with an enhancement in both cell adherence and proliferation [62]. Therefore, the PCL/2%nHA coating is more desirable than pure PCL coating in biomedical application.

#### 2.1.5. Crystallization Kinetics and Thermal Stability of Mg–Zn–Ca MGs

The crystallization behavior of MGs has attracted tremendous attention from researchers. On the one hand, it contributes to investigate deformation behaviors at high temperature, determines the appropriate range of processing temperature, and clarifies the GFA. On the other hand, the stability of glasses can be improved on the basis of the relation between crystallization kinetics and alloy compositions, etc. [77]. There have been many reports on the crystallization kinetics and thermal stability of MGs. Hu et al. [77] investigated the crystallization behavior of Ca_65_Mg_15_Zn_20_ MG and confirmed that the crystallization of Ca_65_Mg_15_Zn_20_ MG was governed by diffusion-controlled, three-dimensional growth. After isothermal annealing, the crystallization product was detected to be CaMg_2_, with a particle size of about 5 nm. Sun et al. [78] have confirmed that the major phase in the initial crystallization of Mg_61_Cu_28_Gd_11_ MG was the Mg_2_Cu phase. For (Mg_61_Cu_28_Gd_11_)_98_Cd_2_ MG, the precipitation of the primary Mg_2_Cu phase was suppressed by the addition of Cd, which enhanced the resistance to the formation of the Mg_2_Cu phase and, thus, improved the thermal stability. This was explicable by the strong affinity between Cd–Mg and Cd–Gd, as well as the larger difference between Cd and the main constitutes from the perspective of overall atomic size [78].

Zhang et al. [79] clarified the crystallization process of Ca_4_Mg_72-x_Zn_24+x_ (x = 0–12, ∆x = 2 at.%) MGs. At lower temperatures (390 to 400 °C), the crystallization was initiated by the precipitation of Mg_51_Zn_20_ crystals. During the second crystallization event, the Ca_16.7_Mg_38.2_Zn_45.1_ ternary compound and Mg-hcp precipitated from the residual amorphous phase. Subsequently, the formation of Ca_1.5_Mg_55.3_Zn_43.2_ ternary compound happened at higher temperature. Finally, the crystallization process terminated via Ca_1.5_Mg_55.3_Zn_43.2_, transforming to Ca_2_Mg_5_Zn_13_ before melting. Furthermore, with the maximum T_rg_ and activation energy, the Ca_4.2_Mg_68.7_Zn_27.1_ MG exhibits the best GFA and thermal stability in the composition range of Ca_4_Mg_72-x_Zn_24+x_ MGs. Zhang et al. [80] realized the controlling phase transitions in Mg_65_Zn_30_Ca_5_ MG by nanocalorimetry. An underlying intermediate amorphous phase was detected at heating rates higher than 4000 K/s. With a reduced reaction temperature and lower activation energy, the formation of the Mg_7_Zn_3_ phase was facilitated by this metastable phase. Opitek et al. [81] studied the crystallization process of Mg_72_Zn_24_Ca_4_ MG. They found that the GFA and crystallization were both significantly influenced by the heating rate, and the crystallization process was governed by diffusion-controlled grain growth. Additionally, they confirmed that the Mg_72_Zn_24_Ca_4_ MG was stable at the human body temperature (36.6 °C) [81].

### 2.2. Mechanical Properties

**Figure 2:**
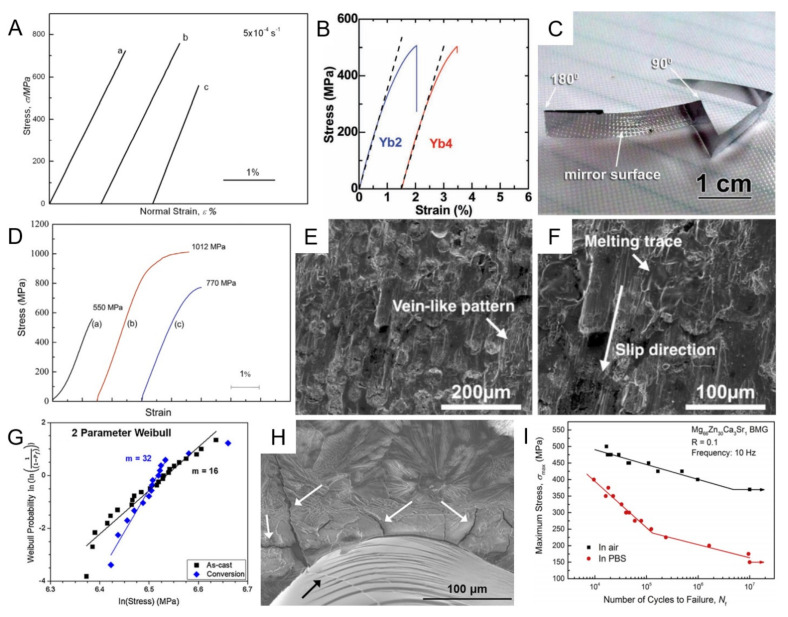
Mechanical properties of Mg–Zn–Ca-based MGs. (**A**) Compressive curves of as-cast Mg_65_Zn_30_Ca_5_, Mg_65_Zn_30_Ca_4_Ag_1_, and Mg_63_Zn_30_Ca_4_Ag_3_ alloy rods (2 mm in diameter) under a strain rate of 5 × 10^−4^ s^−1^, reproduced with permission from [83]. (**B**) The plots of tensile stress versus strain curve at strain rate of 10^−4^ s^−1^ for the Yb2 and Yb4 MG ribbons, reproduced with permission from [84]. (**C**) The optical image of bent Yb2 MG ribbon, reproduced with permission from [84]. (**D**) Engineering strain–stress curves of the Mg_69−x_Zn_27_Ca_4_Y_x_ alloys: (a) x = 0, (b) x = 1, and (c) x = 2 at.%, reproduced with permission from [59]. (**E**) The vein-like pattern and (**F**) the melting trace and the slip direction of the fracture surface of Mg_66_Zn_29_Ca_5_ MGMC with 10 vol% of porous Mo particles, reproduced with permission from [74]. (**G**) Fitted 2 parameter Weibull statistics for the fracture strength of as-cast and PCC compression rods and their corresponding fitted shape parameters (m), reproduced with permission from [89]. (**H**) A micrograph of a conversion-coated sample, following failure under compression (white arrows indicated the coating spalling, cracking, and delamination along the fracture, and black arrow indicated further shear band and crack formation), reproduced with permission from [89]. (**I**) Stress-life curves for the compression–compression fatigue tests in air and in PBS of Mg_66_Zn_30_Ca_3_Sr_1_ MG, reproduced with permission from [95].

Mg–Zn–Ca MGs possessed high specific strength of 250–300 MPa cm^3^/g [21], as well as an appropriate mass density, ranging from 2.0 to 3.0 g/cm^3^, which is close to the ideal value of biodegradable orthopedic implants (1.8–2.0 g/cm^3^) [21,45]. The elastic modulus of the Mg–Zn–Ca MGs also closely matched those of the cortical bone [82]. Furthermore, compared to pure Mg with a compression fracture strength of 198.1 ± 4.5 MPa, the Mg_70_Zn_25_Ca_5_ and Mg_66_Zn_30_Ca_4_ MGs exhibited much higher fracture strength (565.8 ± 23.2 and 531.2 ± 22.8 MPa, respectively) [82]. As displayed in Figure 2A, the deformation of the Mg_65_Zn_30_Ca_5_ MG and the MG with minor additions of Ag happened through elastic deformation. All of those alloys were easy to break into pieces during testing with almost zero plasticity, which indicated that the Mg–Zn–Ca MGs were brittle materials, or at least “macroscopically brittle” [83]. Zhao et al. [63] demonstrated that the dependability of fracture strength of Mg–Zn–Ca MGs was higher than brittle engineering ceramic materials by Weibull statistics analysis, although Mg–Zn–Ca MGs were considered brittle materials. That may be due to the dissipation of some plastic energy in the localized shear bands [18]. However, most bioresorbable implants do not allow such low plasticity because they will be placed under stress from the body, and shattering would be catastrophic. To this end, minor additions and Mg–Zn–Ca MGMCs have been adopted to deal with it. Yu et al. [84] significantly improved the ductility of Mg–Zn–Ca MG for the first time, through microalloying 2~4 at.% rare-earth Yb elements (Figure 2B). The bending test, displayed in Figure 2C, further revealed the modified ductility of Mg_66_Zn_30_Ca_4_ MG, through Yb additions, where the Yb2 ribbon with a mirror-like surface was not broken even bent for 180° [84]. The increased plasticity was explicable by the reduction in the shear modulus and increase in shear band density after alloying with Yb [84]. Wang et al. [59] also successfully obtained Mg–Zn–Ca MGs-based alloys with improved plastic by addition of minor Y. As shown in Figure 2D, Mg_68_Zn_27_Ca_4_Y_1_ alloy exhibited an enhanced capacity for plastic strain, which was above 3.1% [59]. The improvement in plasticity may be related to the strengthening phases formed in the microstructure after the addition of minor Y. Although it has been improved, the ductility of Mg–Zn–Ca-based MGs still must be improved. The preparation of ex situ MGMCs is another common method to improve the plastic of MGs, in which the added second phases can prevent the evolvement of shear bands to macrocracks and promote the generation of numerous shear bands [85]. For instance, Figure 2E,F displayed fracture surface of Mg_66_Zn_29_Ca_5_ MGMC with porous Mo particles (10 vol.%) and presented that the localized plasticity was derived from the porous Mo particles, which assimilated lots of energies of the shear bands [74]. Besides, surface coating could also improve the ductility of MGs [86,87,88]. Miskovic and co-workers prepared an Mg–Zn–Ca MG coated with a phosphate conversion coating, and they demonstrated that the conversion coating could enhance the mechanical performance of the Mg–Zn–Ca MG [89]. A two-fold increase in mechanical properties and enhancement in minimum fracture strength was achieved (Figure 2G). The thin film contributed to the geometric constraints of the substrate and energy dissipation along the surface under compressive loads (Figure 2H) [89]. To date, there are many studies reporting improvements in the mechanical properties of Mg–Zn–Ca MGs (Table 2) [27,45,58,59,60,68,69,70,71,73,82,83,84,89,90,91,92,93,94]. However, the elongation of reported Mg–Zn–Ca-based MGs is still low and cannot meet the requirement for orthopedic implants, which means material development toward Mg–Zn–Ca-based MGs still has a long way to go, and technical bottlenecks have yet to be overcome.

It is worth noting that the environment is more aggressive inside the human body than that of the air. Li et al. indicated that Mg–Zn–Ca MGs possessed a reduced fatigue life in phosphate-buffered saline (PBS) solution than that of the air above the fatigue endurance limit (Figure 2I) [95]. The compressive strength of Mg–Zn–Ca MGs also decreased seriously in the chemistry-mechanics interactive environments [96]. As is reported, fixation screw must sustain 95% of initial load-bearing capability for more than 6 weeks after implantation [97], and stents should perform properly about 18 months during the cardiovascular intervention [98]. Therefore, the biomedical implant should possess mechanical properties that allow it to withstand the chemical and stress environment in vivo for a long time. Li et al. [95] concluded that Mg_66_Zn_30_Ca_3_Sr_1_ MG met the qualification for biomaterials from the aspect of fatigue property. Song et al. designed a core-shell structure with a Mg crystal core and Mg–Zn–Ca MG shell to restrain intergranular stress corrosion cracking, which resulted in the significant reduction in hardness during degradation [57]. Therefore, this structure could obtain more stable mechanical properties relative to the form of a solid monolith (in a rod or plate form).

## 3. Biocorrosion and Biocompatibility of Mg–Zn–Ca-Based MGs

### 3.1. Biocorrosion

In crystalline and amorphous metallic materials, alloying elements can enter the surrounding environment, due to corrosion (a primary mechanism along with wear), resulting in toxic effects, adverse biological reactions, site accumulation, and, ultimately, implant failure. Thus, in addition to being harmful to mechanical properties, the corrosion process also dictates biocompatibility [99]. Resultantly, excellent biocorrosion resistance is essential for biodegradable implant materials to maintain the required mechanical integrity and suppress the release of metallic ions caused by corrosion during the healing period. Table 3 [26,28,54,55,59,66,68,69,70,71,76,82,83,100,101,102,103,104,105] presents the corrosion rate measured by the electrochemical test. Biological media, such as artificial body fluid, Hank’s solution, minimum essential medial (MEM), PBS, Ringer’s solution, and simulated body fluid (SBF), were used to simulate the human body environment. The biocorrosion behaviour of Mg–Zn–Ca MGs was mostly studied by electrochemical and immersion tests in biological media. Immersion tests usually include hydrogen evolution measurement, ion concentration measurement, pH monitoring, and so on, while electrochemical tests include potentiodynamic polarization (PDP), electrochemical impedance spectroscopy (EIS), etc.

**Figure 3:**
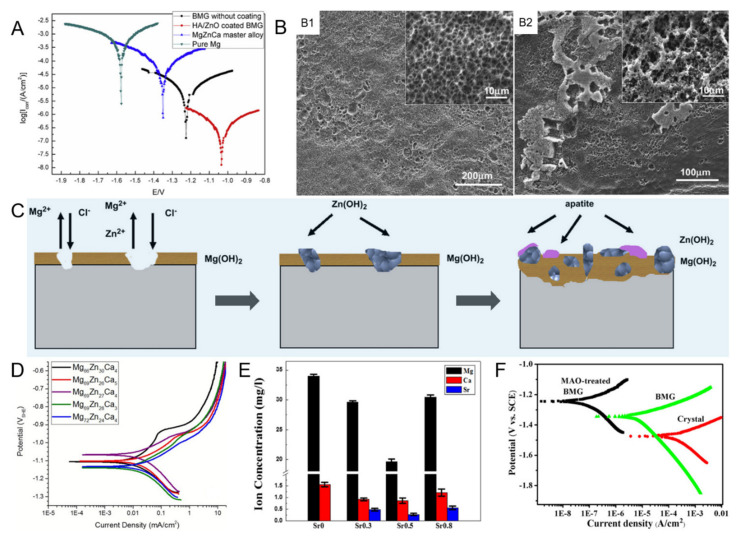
Biocorrosion of Mg–Zn–Ca-based MGs. (**A**) Polarization curves of Mg, Mg_68_Zn_28_Ca_4_ MG, crystal Mg_68_Zn_28_Ca_4_ alloy, and HA-coated MG, reproduced with permission from [61]. (**B**) SEM images of the surface morphologies of (**B1**) Mg_66_Zn_30_Ca_4_ and (**B2**) Mg_70_Zn_25_Ca_5_ MGs after immersing in CrO_3_ solution for 10 min, reproduced with permission from [82]. (**C**) The sketch map for the evolution of corrosion process of Mg–Zn–Ca MG immersed in SBF, reproduced with permission from [82]. (**D**) Representative polarisation curves of Mg rich MGs in MEM at 37 °C and 5% CO_2_, reproduced with permission from [102]. (**E**) Metallic ion concentrations of the solution after the 3-days immersion test in PBS at 310 K, reproduced with permission from [66]. (**F**) Polarization curves in SBF of Mg_65.2_Zn_28.8_Ca_6_ crystalline alloy, Mg_65.2_Zn_28.8_Ca_6_ MG, and MAO-treated Mg_65.2_Zn_28.8_Ca_6_ MG, reproduced with permission from [76].

Mg–Zn–Ca MGs exhibited higher corrosion resistance than corresponding crystalline Mg alloys [82] for two reasons. The first reason is that Mg–Zn–Ca MGs possess the microstructure with no second phases and free of microstructural defects (such as grain boundaries and dislocations) that could be the unsubstantial areas for etching initiation. With the lack of microstructural defects, galvanic couples are reduced, which prevents intergranular corrosion. Additionally, the absence of structural defects can suppress ion diffusion, improving corrosion resistance [106]. For instance, Mg_70_Zn_25_Ca_5_ MG showed a more even corrosive morphology than that of as-deformed pure Mg, attributed to the homogeneous structure that minimizes galvanic corrosion [82]. Zhou et al. [104] found that the corrosion resistance of Mg_68_Zn_28_Ca_4_ MG was much higher than that of the crystalline equivalent (Figure 3A), due to compact structure, uniform composition, and the fact that it is free of defects of MGs. Furthermore, because of destroyed amorphous structure by micro-alloying of Y or Mn, the alloys after microalloying exhibited higher corrosion resistance than pure Mg, but lower than Mg–Zn–Ca MGs without the addition of Y or Mn [59,68,107]. Another striking aspect that makes Mg–Zn–Ca MGs highly corrosion-resistant systems is the wide composition space, which allows for the concentration of Zn to be adjusted high enough to form protective oxide layers. For instance, Zberg et al. [25] revealed that a O- and Zn-rich passivation layer was formed on the Zn-rich (≥28 at.%) alloy surface, which can be ascribed to the expanding solubility of Zn in the amorphous system. The passivation layer can preserve the surface and result in the release of only a small amount of hydrogen during in vivo and in vitro degradation. The corrosion mechanism of Mg–Zn–Ca MGs changed with Zn content over 28 at.% [25]. As shown in Figure 3B, Mg_66_Zn_30_Ca_4_ and Mg_70_Zn_25_Ca_5_ MGs exhibited different corrosion behaviour, which further demonstrated the change of corrosion mechanism when Zn content exceeded 28 at.% in Mg–Zn–Ca MGs. Gu et al. [82] proposed the corrosive mechanism of the Mg–Zn–Ca MG immersed in SBF (Figure 3C). The formation of ZnO/Zn(OH)_2_ played a vital role in protecting the surface. The rapid dissolution of Sr resulted in the rise of local pH, leading to the rapid deposition of Zn(OH)_2_, which made the corrosion resistance of Mg–Zn–Ca–Sr MGs stronger than the Sr-free Mg–Zn–Ca MG [58]. Sun et al. [108] proved that the alloying of Sr into Mg_66_Zn_30_Ca_4_ MG reduced the p and s orbital states of surface Zn and Mg elements near the Fermi level, effectively suppressing the electron transfer and increasing the surface corrosion resistance of Mg_66_Zn_30_Ca_4_ MG. Figure 3D presented some representative polarisation curves of Mg–Zn–Ca MGs. The lowered anodic dissolution and presence of a shoulder, as displayed in Figure 3D, was ascribed to the increased Zn content of the alloys [102].

Extensive efforts have been devoted to improving the biocorrosion resistance of Mg–Zn–Ca MGs (Table 3). Wang et al. found that the addition of 0.5 at.% Sr markedly enhanced the biocorrosion resistance of Mg–Zn–Ca–Mn alloys, which could be ascribed to the generation of the effective defense of the uniform Zn(OH)_2_ sediment layer on the MG surface [66]. The appropriate amount of Sr could restrain the precipitation of the MnZn_13_ dendrites [66], which improved the microstructure. Besides, the highest Mg^2+^ ion concentration was still no more than the daily absorption limit of the body (Figure 3E) [66]. The minor addition of 1 at.% Ag could improve the polarization resistance, but further addition of 3 at.% Ag reduced corrosion resistance. This reduction was related to the destroyed amorphous structure [83]. However, it is interesting that, although the matrix of the Mg–Zn–Ca MGMC was crystallized, due to the addition of porous NiTi particles, the composite exhibited modified corrosion resistance than the monolithic Mg–Zn–Ca MG [60]. Because the existence of NiTi dispersions with excellent corrosion resistance among the matrix could suppress the corrosion process and effectively decrease the corrosion rate [60]. Chen et al. [76] prepared a silicon-containing film on the Mg_65.2_Zn_28.8_Ca_6_ MG by MAO treatment. The polarization curves (Figure 3F) demonstrated that the corrosion resistance of the Mg–Zn–Ca MG was significantly improved by this surface treatment. Compared to the bare Mg–Zn–Ca MG, the corrosion potential of MAO-treated MG was increased by 101 mV, and the corrosion density was decreased by two orders [76]. The improved etching resistance could be attributed to the thick and dense inner coating and a large amount of amorphous phase in the coating, which can hinder the corrosion process [76]. Furthermore, the immersion testing revealed that the MAO coating could encourage the formation of apatite, which could fill the micropores on the porous outer layer, thus preventing the corrosive ions (such as Cl^−^) from going into coatings [76].

### 3.2. Biocompatibility

Biocompatibility refers to the ability of a material to respond appropriately to the host in a particular application [109]. The component elements of the Mg–Zn–Ca MGs are bio-safe, implying the biocompatibility of implants. In vivo histopathology analyses and in vitro cytotoxicity tests are often used to rate the biocompatibility of Mg–Zn–Ca MGs. In vitro cytotoxicity tests include indirect cytotoxicity tests, in which cells are cultured in materials extraction mediums, and direct cytotoxicity tests, in which cells are cultured on materials directly. Additionally, the cell exhibited reduced viability in direct cytotoxicity tests because cells are sensitive to the environment fluctuation (hydrogen evolution, corrosion product, etc.), and the influencing factors would increase in direct cytotoxicity tests [110].

#### 3.2.1. Cellular Biocompatibility

**Figure 4:**
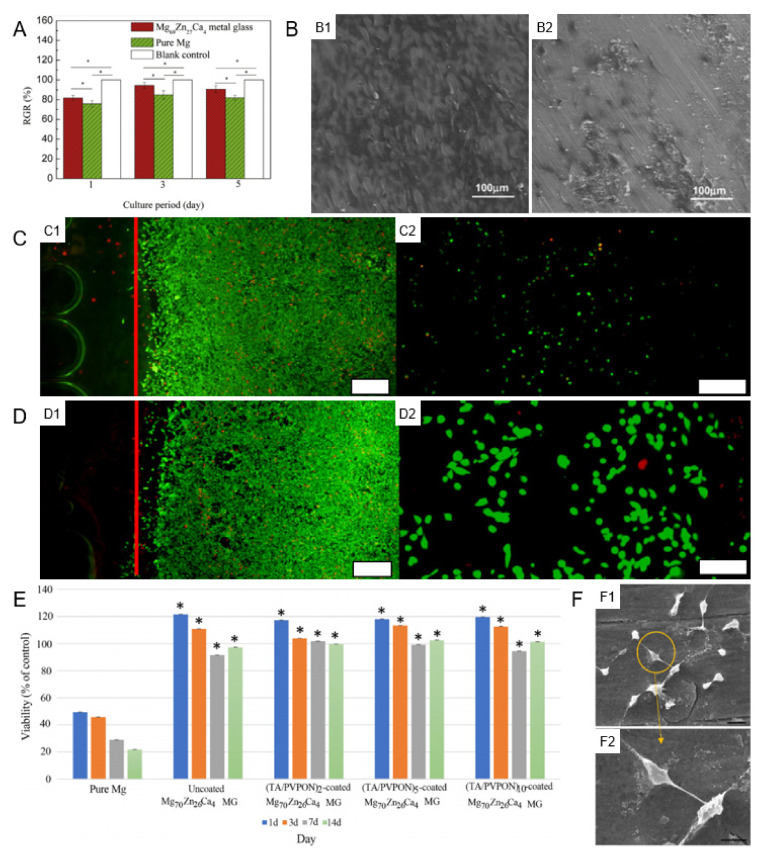
Cellar biocompatibility of Mg–Zn–Ca-based MGs. (**A**) Cell viability after incubation with different extracts for 1, 3, and 5 days, * *p* < 0.05, reproduced with permission from [54]. (**B**) The morphology of MG63 cells cultured on (**B1**) Mg_66_Zn_30_Ca_4_ and (**B2**) Mg_70_Zn_25_Ca_5_ MG samples for 5 days, reproduced with permission from [82]. (**C**) Live (green)/dead (red) cell staining of attached MG63 cells around amorphous Mg_67_Zn_28_Ca_5_ alloy without coating at (**C1**) 40× and (**C2**) 50× magnification, reproduced with permission from [112]. (**D**) Live (green)/dead (red) cell staining of attached MG63 cells around amorphous Mg_67_Zn_28_Ca_5_ alloy with gelatin coating/2-day crosslinking at (**D1**) 40× and (**D2**) 50× magnification. White bar = 100 μm, reproduced with permission from [112]. (**E**) MTT assay results for coated and uncoated Mg_70_Zn_26_Ca_4_ MG ribbon and pure Mg (* *p* < 0.05, compared to pure Mg), reproduced with permission from [114]. (**F**) SEM images of Schwann cell morphology on the surfaces of Mg_70_Zn_26_Ca_4_ MG ribbon. (**F1**) Black bar = 10 μm. (**F2**) Black bar = 5 μm, reproduced with permission from [114].

Chen et al. demonstrated that Mg_69_Zn_27_Ca_4_ MG possessed better cell viability, as compared to pure Mg via indirect cell cytotoxicity tests (Figure 4A) [54] because the extract of the Mg_69_Zn_27_Ca_4_ MG contained more nutritious elements (Mg^2+^, Zn^2+^, and Ca^2+^) and showed a lower pH value than that of pure Mg extract, which was more conducive to cell growth [54,111]. Gu et al. proved that Mg_66_Zn_30_Ca_4_ and Mg_70_Zn_25_Ca_5_ MGs all exhibited good cell viability, and Mg_66_Zn_30_Ca_4_ MG showed better cell adhesion and viability than Mg_70_Zn_25_Ca_5_ MG (Figure 4(B1,B2)) [82]. It can be seen from Figure 4(B2) that there were some micro-cracks on the Mg_70_Zn_25_Ca_5_ MG after culture for 5 days, and sometimes materials were even broken into several parts, which was not conducive to cell proliferation [82]. Figure 4(C1,C2) displayed that the cell was high viable around the alloys, but there was poor adhesion and survival on the alloys. To settle this, Chan et al. [112] prepared a gelatin coating by electrospinning on Mg_67_Zn_28_Ca_5_ MG. The gelatin layer, which hydrolyzes easily, was then crosslinked by the dehydrothermal (DHT) method for 2 or 5 days. Gelatin is a hydrolyzed collagen and possesses good bioactivity to improve the adhesion of many kinds of cells. As displayed in Figure 4(D1,D2), gelatin-coating, with 2 days of DHT crosslinking, significantly improved the adhesion of viable cells. Mg–Zn–Ca–Sr MGs exhibited good cytocompatibility, as the healthy and well-adhered cells were seen on the MG surface [58]. In addition, Sr has been reported to promote bone cell replication and protein synthesis, as well as depress bone resorption [113]. Mg–Zn–Ca–Ag MGs showed higher cytocompatibility than the Ag-free Mg–Zn–Ca MG, and the amount of Ag released could be very low, suggesting the effect of Ag on cell behavior may be limited [71].

It is worth noting that all of the above-mentioned cytocompatibility studies are focused on orthopedic implants. Moreover, the cytocompatibility of Mg–Zn–Ca MGs is not limited to orthopedic cells. For instance, Figure 4E presented Schwann cells, which play a vital role in nerve tissue reconstruction, and possessed better viability for Mg_70_Zn_26_Ca_4_ MG ribbon extract than that of pure Mg extract at all time-points, revealing the good cytocompatibility of the Mg_70_Zn_26_Ca_4_ MG [114]. Figure 4F presented the proliferation and adhesion of Schwann cells on the surface of Mg_70_Zn_26_Ca_4_ MG, with a typical Schwann cell morphology, which proved cells were healthy [114]. More different types of cytocompatibility experiments are worth carrying out in the future.

#### 3.2.2. Tissue Biocompatibility

**Figure 5:**
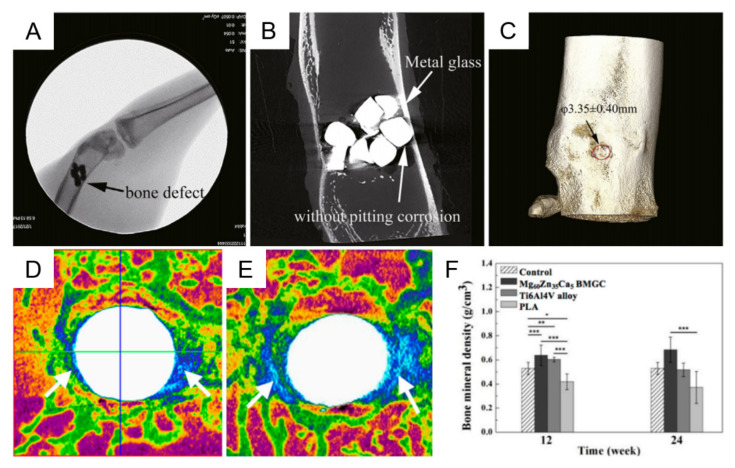
X-ray (**A**), μ-CT (**B**), and 3D reconstruction photographs (**C**) of Mg_69_Zn_27_Ca_4_ MG at 2 months postoperation, reproduced with permission from [54]. Micro-CT image of the rabbit’s femur implanted with Mg_60_Zn_35_Ca_5_ MGMC at 12 (**D**) and 24 (**E**) weeks postoperatively. (**F**) Intergroup comparison of bone mineral density surrounding the implanted site at 12 and 24 weeks, analyzed with CTan analyzer software (* *p* < 0.05, ** *p* < 0.01, *** *p* < 0.001) [116].

Chen et al. carried out histopathology evaluation by implanting Mg_69_Zn_27_Ca_4_ MG and β-TCP into the right and left legs of rabbits, respectively [54]. The formation of new bone in the Mg_69_Zn_27_Ca_4_ MG implant was in compact contact with the implant at the cortical and medullary cavity site (Figure 5A), while there was an obvious cave in the β-TCP (commonly bone substitute in the clinic) implant, with no marked relationship between the implant and the tissue. The μ-CT results, displayed in Figure 5B, suggested Mg–Zn–Ca MG exhibited high corrosion resistance and, thus, suppressed the gas cavity formation and osteolysis ascribed to a high degradation rate of Mg alloys. In comparison with the φ4.14 mm unhealed hole of bone defects, the size of the unhealed hole was only φ3.35 mm in the group of Mg–Zn–Ca MG (Figure 5C) [54,115]. This result implied that the healing effect of Mg–Zn–Ca MG was better than that of β-TCP. Figure 5D,E showed that noticeable new bone has been formed around the rod after the implantation of Mg_60_Zn_35_Ca_5_ MGMC for 12 and 24 weeks, respectively. Furthermore, the comparison of bone mineral density (BMD) around the implantation site revealed that the levels of BMD (*p* < 0.001) in the Mg_60_Zn_35_Ca_5_ MGMC and Ti6Al4V alloy groups (implantation for 12 and 24 weeks) were obviously higher than those in the control and PLA groups (Figure 5F). Although the BMD reduced from week 12 to week 24 in the Ti6Al4V alloy group, the decline in BMD was not pronounced for the Mg_60_Zn_35_Ca_5_ MGMC group (Figure 5F), suggesting a more sustainable osteo-promoting effect after in situ release of Mg ions [116].

**Figure 6:**
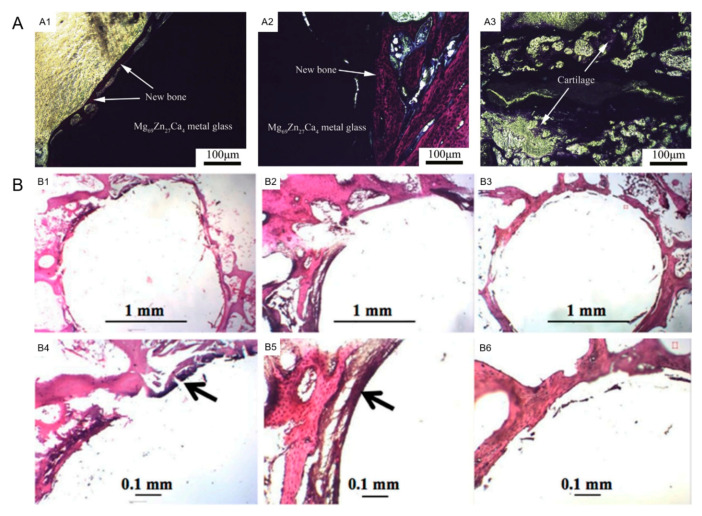
Tissue biocompatibility of Mg–Zn–Ca-based MGs. (**A**) VG photographs of bone defect repair for 2 months postoperation: (**A1**,**A2**) Mg_69_Zn_27_Ca_4_ MG, (**A3**) β-TCP (the red parts: new bone, the purple parts: cartilage), reproduced with permission from [54]. (**B**) Histological images of the implanted site at 24 weeks. (**B1**,**B4**) Mg_60_Zn_35_Ca_5_ MGMC; (**B2**,**B5**) Ti6Al4V alloy; (**B3**,**B6**) PLA. Black arrows indicate new bone formation (hematoxylin and eosin staining) [116].

As shown in Figure 6(A1), the new bone formed around the Mg_69_Zn_27_Ca_4_ MG, without obvious adverse tissue reactions around the implants. It was also found that a cancellous bone adhered closely to the MG implants with progressing implantation time, whereas only some cartilages formed around the β-TCP implants (Figure 6(A2,A3)). This suggested that the osteogenesis ability of Mg–Zn–Ca MG was superior, compared to β-TCP in the early implantation (2 months) [54]. Similarly, Zebrg et al. [25] found that there was no obvious hydrogen evolution around Mg_69_Zn_27_Ca_4_ MG. They also concluded that Zn-rich (≥28 at.%) Mg–Zn–Ca MGs would release much less hydrogen than Mg alloys in vivo. No inflammatory reaction was observed for the implants, suggesting that Zn-rich Mg–Zn–Ca MGs possessed good biocompatibility. Wong et al. [116] also confirmed that the Mg_60_Zn_35_Ca_5_ MGMC with 40 vol.% Ti particles exhibited better performance, over the traditional Ti6Al4V alloy and PLA, in the osteogenic and osteoconductive aspects. As displayed in Figure 6B, obvious new bone formed, surrounding the Mg–Zn–Ca MGMC and Ti6Al4V alloy, after 24 weeks implantation. With the smooth interface between the bone tissue and the PLA implants, the formation of the new bone was very limited. Additionally, the bone tissue of the Mg–Zn–Ca MGMC implants presented smoother interface morphology, as well as a much denser bone matrix, compared to those of Ti6Al4V alloy and PLA implants, implying that the Mg–Zn–Ca MGMC had the best osteo-promoting effects [116]. Above all, in vitro cytotoxicity test and in vivo histopathology analysis suggested that Mg–Zn–Ca-based MGs possessed high cell viability and good osteogenesis activity. Consequently, Mg–Zn–Ca-based MGs were proven excellent in biocompatibility.

## 4. Conclusions and Outlook

At present, the critical diameter (d_c_) of Mg–Zn–Ca MGs is still limited to millimeters, due to their limited GFA. As for mechanical properties, while the brittleness of Mg–Zn–Ca MGs has been significantly improved by some methods, it still cannot meet the requirements of orthopedic implants. Consequently, the application of Mg–Zn–Ca MGs for biomedical implant remains in the early stage. To promote the development of Mg–Zn–Ca MGs, for clinical application, the following aspects present great potential.

Fabrication routes: Conventional Mg–Zn–Ca MGs are fabricated by the induction-melting/copper mold injection or melt spinning method and have insufficient size, due to the limited GFA. Moreover, the microstructure of the achieved samples is usually not completely amorphous with increasing of the size. The 3D printing technology, belonging to a cutting-edge, bottom-up preparation method, can theoretically free MGs from the size and geometry restrictions in as-cast specimens; thus, a large size of MGs, with complex geometries, can be obtained. Currently, selective laser melting (SLM) is the most commonly adopted 3D printing techniques for producing MGs. Fe-, Al-, Zr-, Cu-, and Ti-based MG systems, which have been produced by SLM technique [117]. However, research on Mg–Zn–Ca MGs fabricated by 3D printing techniques has rarely been carried out. Therefore, to break through the limited size of Mg–Zn–Ca-based MGs, 3D printing techniques are worth trying out. The two problems of material oxidation and defect control in the 3D printing process need to be solved simultaneously.

Composition design: So far, the elements that have been introduced into the Mg–Zn–Ca MG systems include Li, Sr, Mn, Y, Ag, Cu, Ga, Au, Pd, Yb, Nb, etc. The effects of these elements on the structure and properties of Mg–Zn–Ca MGs have been studied. However, other elements that also meet the biodegradability and biocompatibility criteria, such as K, Na, Rb, Sn, Ba, Cs, Mo, Sc, and W [118], have not been added in Mg–Zn–Ca-based MG systems. Their effects toward Mg–Zn–Ca-based MGs are still unknow. Relevant research is worth carrying out in the future.

Structure design: In predominant studies, Mg–Zn–Ca-based MGs are usually studied in the form of a rod or plate. A novel structure may bring change to the current status. The designed structure should reduce the dependency of d_c_, while maintaining the large size and mechanical strength. For instance, a hollow cylindrical scaffold structure has been applied in Mg alloys. It was designed to reduce the used mass of the alloy for implantation in a shape imitating a cortical bone. In particular, this structure possesses a hollow cylinder shape. The internal open space will facilitate the removal of degradation byproducts and the in-growth of tissue [119]. Furthermore, the mechanical properties and biocorrosion resistance of Mg–Zn–Ca-based MGs should be balanced when designing the alloy structure.

In addition to the above macro structural design, novel micro structural design strategy can significantly improve the overall properties of materials. Recently, nanoglasses with rich glass–glass interfaces and nanostructured dual-phase metallic glasses (DP-MGs) were designed and expected to be new strategies for preparing high-performance MGs. For instance, the as-developed metallic glass/oxide glass nanocomposite, with continuous glass–glass interfaces, possessed a supra-nanometer-sized dual-phase structure, enabling an obvious tensile plasticity of 2.7% [120]. A plastic strain of 15% under uniaxial tension could even be achieved by the Sc_75_Fe_25_ nanoglass with rich glass–glass interfaces [121]. Furthermore, nanostructured Mg–Zn–Ca DP-MGs (10 nm-wide amorphous phases embedded in amorphous matrix) were developed. The 10 nm-wide amorphous phases allow for oxygen propagation into the DP-MG, resulting in a micrometer-thick hydroxides–oxides layer and inducing a dramatically reduced corrosion rate (77% lower than that of pure Mg) in SBF [122]. These results shed light on a helpful approach to improve the plasticity and corrosion resistance of Mg–Zn–Ca-based MGs by constructing novel nanostructures. New nanostructure-building strategies toward Mg–Zn–Ca-based MGs are scarce and need extensive development.

Reinforcement approaches: Metallic glass matrix composites (MGMCs) and surface modification or coating are two common approaches that were adopted to ameliorate the brittle behavior and corrosion resistance of Mg–Zn–Ca MGs. At present, there are many reports that showed significant improvements by these approaches, but the research that combines the two technique is lacking. It is important to combine these two methods because Mg–Zn–Ca MGs require both good mechanical properties and biocorrosion resistance for biomedical implant application.

The mainstream strategy to ameliorate the brittle behavior of Mg–Zn–Ca MGs is to introduce a softer crystal structure in the MG, which not only hinders the reproduction and spread of shear bands, but also promotes multiple shear bands, inducing monolithic deformation throughout the MG composite. However, the addition of ex situ phases or formation of in situ crystalline phases can destroy the amorphous structure of Mg–Zn–Ca MGs, leading to worse GFA and reduced corrosion resistance. Mg–Zn–Ca-based MGs with a minor addition of certain elements (such as Sr, Ga) exhibit an improved GFA, but their plasticity is still almost zero. Therefore, there is a need for a new approach that can enhance the ductile properties, without affecting the amorphous structure of materials.

Biodegradable Mg–Zn–Ca-based MGs are promising as temporary implant materials, due to their excellent biocompatibility, high strength, appropriate Young’s modulus, and superior corrosion resistance, compared to crystalline Mg. By using some strategies, including minor alloying additions, in situ or ex situ second ductile phase reinforced MG matrixes, and surface coating strategies, biodegradable Mg–Zn–Ca-based MGs are one step closer to clinical use, particularly in orthopedic implants. In vitro and in vivo tests have also validated the advantages of Mg–Zn–Ca-based MGs, with high cell viability and good osteogenesis activity. However, efforts are still required to overcome the existing challenges, before final successful clinical applications.

## Figures and Tables

**Table 1:** Mechanical properties of Mg–Zn–Ca MGs.

Materials (at.%)	d_c_ (mm)	σ_f_ (MPa)	R_m_ (MPa)	σ_y_ (MPa)	A (%)	H_v_ (HV)	Ref
Mg_60_Zn_34_Ca_6_	3	—	—	888	—	296 ± 25	[55]
Mg_62_Zn_32_Ca_6_	2	—	110	—	0	218	[28]
Mg_63_Zn_32_Ca_5_	3	—	156	—	0.2	261	[28]
Mg_64_Zn_30_Ca_6_	2	—	160	—	0.2	244	[28]
Mg_64_Zn_32_Ca_4_	3	—	166	—	0.6	263	[28]
Mg_65_Zn_30_Ca_5_	3	—	191	—	0	284	[28]
Mg_65_Zn_32_Ca_3_	1	—	175	—	0.2	272	[28]
Mg_66_Zn_28_Ca_6_	2	—	90	—	0	212	[28]
Mg_66_Zn_30_Ca_4_	5	716–854	—	—	—	—	[63]
Mg_66_Zn_30_Ca_4_	4	787 ± 22	—	—	—	2.45 ± 0.01 (GPa)	[58]
Mg_66_Zn_30_Ca_4_	4	—	191	—	0.3	291	[28]
Mg_67_Zn_28_Ca_5_	2	622	—	662	0.2 ^1^	—	[27]
Mg_67_Zn_28_Ca_5_	3	—	117	—	0.6	233	[28]
Mg_67_Zn_30_Ca_3_	1	—	195	—	0.2	305	[28]
Mg_68_Zn_28_Ca_4_	3	671	—	540	0.43 ^1^	—	[27]
Mg_68_Zn_28_Ca_4_	4	—	125	—	0.1	235	[28]
Mg_69_Zn_28_Ca_3_	2	675	—	591	0.5 ^1^	—	[27]
Mg_69_Zn_28_Ca_3_	1	—	128	—	0.1	237	[28]
Mg_71_Zn_25_Ca_4_	≥2	672–752	—	—	—	—	[63]
Mg_73_Zn_23_Ca_4_	2	—	—	636	—	212 ± 19	[55]
Mg_80−x_Ca_5_Zn_15+x_ (x = 5–20)	1–4	700	—	—	—	2.16 (GPa)	[21]

σ_f_: Compressive fracture strength, R_m_: highest tensile strength, σ_y_: compressive yield strength, A: elongation, H_v_: microhardness, ^1^ plastic strain. All samples were prepared by copper mould injection-casting method.

**Table 2:** Mechanical properties of Mg–Zn–Ca-based MGs.

Materials (at.%)	D (mm)	σ_f_ (MPa)	A (%)	E (GPa)	H_v_ (GPa)	Ref
Desirable materials for orthopedic implants	Large size	≥230	≥15,10	17–22	—	[45]
Mg_60_Zn_35_Ca_5_	2	571	—	—	—	[90]
Mg_62.9_Zn_32.3_Ca_4.8_	2	590 ± 5.1	—	—	—	[91]
Mg_65_Zn_30_Ca_5_	2	722	—	49	—	[83]
Mg_66_Zn_30_Ca_4_	4	787 ± 22	—	48.8 ± 0.1	2.45 ± 0.01	[58]
Mg_66.2_Zn_28.8_Ca_5_	2	787	—	—	—	[69]
Mg_67_Zn_28_Ca_5_	100 μm (thin wires)	675–894 ^1^	3–5	—	2.16	[92]
Mg_69_Zn_27_Ca_4_	1.5	550 ^2^	1.3	—	—	[59]
Mg_70_Zn_25_Ca_5_	2	565.8 ± 23.2	—	—	—	[82]
Mg_72_Zn_23_Ca_5_	2	—	—	50.38	2.71	[70]
Mg_64_Li_2_Zn_30_Ca_4_	30 μm in thickness	—	—	42.893	1.64	[93]
Mg_63_Li_3_Zn_30_Ca_4_	30 μm in thickness	—	—	54.357	1.98	[93]
Mg_62_Li_4_Zn_30_Ca_4_	30 μm in thickness	—	—	51.541	2.26	[93]
Mg_61_Li_5_Zn_30_Ca_4_	30 μm in thickness	—	—	62.451	3.05	[93]
Mg_68.5_Zn_27_Ca_4_Mn_0.5_	1.5	475	—	—	—	[68]
Mg_68_Zn_27_Ca_4_Mn_1_	1.5	364	—	—	—	[68]
(Mg_66.2_Zn_28.8_Ca_5_)_99_Cu_1_	2	811	—	—	—	[69]
(Mg_66.2_Zn_28.8_Ca_5_)_97_Cu_3_	2	979	—	—	—	[69]
(Mg_66.2_Zn_28.8_Ca_5_)_95_Cu_5_	2	583	—	—	—	[69]
Mg_66_Zn_30_Ca_3.5_Sr_0.5_	6	827 ± 21	—	48.5 ± 0.2	2.49 ± 0.01	[58]
Mg_66_Zn_30_Ca_3_Sr_1_	6	848 ± 21	—	49.1 ± 0.2	2.51 ± 0.02	[58]
Mg_66_Zn_30_Ca_2.5_Sr_1.5_	4	841 ± 24	—	49.4 ± 0.2	2.51 ± 0.02	[58]
Mg_68_Zn_27_Ca_4_Y_1_	1.5	1012 ^2^	3.1	—	—	[59]
Mg_67_Zn_27_Ca_4_Y_2_	1.5	770 ^2^	2.0	—	—	[59]
Mg_65_Zn_30_Ca_4_Ag_1_	2	759	—	50	2.35 ± 0.03	[71,83]
Mg_63_Zn_30.2_Ca_4.5_Ag_2.3_	2	506.5 ± 7.5	—	—	—	[91]
	3	347.6 ± 8.2	—	—	—	
Mg_63_Zn_30_Ca_4_Ag_3_	2	540	—	63	2.35 ± 0.03	[71,83]
Mg_59.8_Zn_33.1_Ca_4.7_Nd_2.4_	2	465.5 ± 6.4	—	—	—	[91]
	3	298.4 ± 9.3	—	—	—	
Mg_66_Zn_30_Ca_2_Yb_2_	40 μm in thickness	500	—	35	—	[84]
Mg_59.3_Zn_32.4_Ca_4.8_Yb_3.5_	2	606.2 ± 4.9	—	—	—	[91]
	3	540.8 ± 5.2	—	—	—	
Mg_66_Zn_30_Yb_4_	40 μm in thickness	500	—	35	—	[84]
Mg_70_Zn_23_Ca_5_Pd_2_	2	—	—	64.20	3.56	[70]
Mg_66_Zn_23_Ca_5_Pd_6_	2	—	—	72.98	3.90	[70]
Mg_60_Zn_35_Ca_5_ MGMC with 50 vol % 20–75 µm Ti particles	2	1190	—	—	—	[73]
Mg_60_Zn_35_Ca_5_ MGMC with 40 vol% spherical Ti particles of 75–105 μm in diameter	2	807	—	—	—	[90]
Mg_67_Zn_28_Ca_5_ MGMC with 40% volume fraction Ti particles of 75–105 μm diameter	2	690	—	—	—	[90]
Mg_67_Zn_29_Ca_4_/NiTi composite	2	~592 ± 22	—	—	—	[60]
Mg_69_Zn_27_Ca_4_/Fe (3 wt% Fe)	1.5	648	1.5	—	—	[94]
Mg_70_Zn_25_Ca_5_ (MGMC)	2	642	—	—	—	[27]
Mg_80_Zn_15_Ca_5_ (MGMC)	3	513	—	—	—	[27]
Mg_66_Zn_30_Ca_4_ with phosphate conversion coated	2	671	—	—	—	[89]

D: diameter, σ_f_: compressive fracture strength, A: elongation, E: Young’s modulus, H_v_: microhardness, ^1^ tensile strength, ^2^ ultimate compress stress.

**Table 3:** Electrochemical corrosion parameters of Mg–Zn–Ca-based MGs.

Materials (at.%)	Electrolyte	E_corr_ (V_SCE_)	i_corr_ (μA/cm^2^)	Corrosion Rate (mm y^−1^)	Ref
Mg_69_Zn_25_Ca_5_Au_1_ (after 1 h immersion)	Artificial physiological fluid	−1.318	25	—	[100]
Mg_69_Zn_25_Ca_5_Cu_1_ (after 1 h immersion)	Artificial physiological fluid	−1.314	63	—	[100]
Mg_69_Zn_25_Ca_5_Au_0.5_Cu_0.5_ (after 1 h immersion)	Artificial physiological fluid	−1.311	57	—	[100]
Mg	Hank’s solution	−1.700 ± 0.050	4.410 ± 0.300	0.100 ± 0.006	[54]
Mg_65_Zn_30_Ca_5_ (ribbon)	Hank’s solution	—	6.6 ^1^	—	[83]
Mg_69_Zn_27_Ca_4_	Hank’s solution	−1.300 ± 0.040	0.440 ± 0.150	0.010 ± 0.003	[54]
Mg_72_Zn_23_Ca_5_	Hank’s solution	—	1.7 (mA cm^−2^)	—	[70]
Mg_70_Zn_23_Ca_5_Pd_2_	Hank’s solution	—	2.1 (mA cm^−2^)	—	[70]
Mg_66_Zn_23_Ca_5_Pd_6_	Hank’s solution	—	2.7 (mA cm^−2^)	—	[70]
Mg_65_Zn_30_Ca_4_Ag_1_ (ribbon)	Hank’s solution	—	3.5 ^1^	—	[83]
Mg_63_Zn_30_Ca_4_Ag_3_ (ribbon)	Hank’s solution	—	19 ^1^	—	[83]
MAO-coated Mg_69_Zn_27_Ca_4_	Hank’s solution	−1.33	0.95	0.31	[101]
Ca-P-coated Mg_69_Zn_27_Ca_4_	Hank’s solution	−1.28	0.31	0.1	[101]
Mg	MEM	−1700 (mV_SHE_)	11.0 (± 6.0)	—	[102]
Mg_65_Zn_30_Ca_5_	MEM	−1.27	6.9	—	[26]
Mg_66_Zn_30_Ca_4_	MEM	–1107 ± 6 (mV_SHE_)	13.1 ± 1.8	—	[102]
Mg_69_Zn_26_Ca_5_	MEM	−1110 ± 6 (mV_SHE_)	16.5 ± 2.3	—	[102]
Mg_69_Zn_27_Ca_4_	MEM	−1083 ± 24 (mV_SHE_)	13.2 ± 2.6	—	[102]
Mg_69_Zn_28_Ca_3_	MEM	−1123 ± 11 (mV_SHE_)	14.4 ± 2.2	—	[102]
Mg_72_Zn_24_Ca_4_	MEM	−1126 ± 25 (mV_SHE_)	19.9 ± 6.0	—	[102]
Mg_66_Zn_30_Ca_4_	PBS	—	—	0.340 ± 0.043 (after the 3-day immersion)	[71]
Mg_66.2_Zn_28.8_Ca_5_	PBS	—	7.41	—	[69]
Mg_69_Zn_27_Ca_4_	PBS	−1.33	10^−4.38^ A/cm^2^	—	[103]
Mg_65.2_Zn_30_Ca_4_Mn_0.8_	PBS	−1.219	104	2.40	[66]
Mg_64.9_Zn_30_Ca_4_Mn_0.8_Sr_0.3_	PBS	−1.1174	34.6	1.32	[66]
Mg_64.7_Zn_30_Ca_4_Mn_0.8_Sr_0.5_	PBS	−1.1173	16.1	0.36	[66]
Mg_64.4_Zn_30_Ca_4_Mn_0.8_Sr_0.8_	PBS	−1.1175	71.8	1.81	[66]
(Mg_66.2_Zn_28.8_Ca_5_)_99_Cu_1_	PBS	—	5.37	—	[69]
(Mg_66.2_Zn_28.8_Ca_5_)_97_Cu_3_	PBS	—	6.91	—	[69]
(Mg_66.2_Zn_28.8_Ca_5_)_95_Cu_5_	PBS	—	60.2	—	[69]
Mg_66_Zn_29_Ca_4_Ag_1_ (after the 3-day immersion)	PBS	—	—	0.308 ± 0.029	[71]
Mg_66_Zn_27_Ca_4_Ag_3_ (after the 3-day immersion)	PBS	—	—	0.265 ± 0.042	[71]
Mg_62_Zn_32_Ca_6_ (after 15 min immersion)	Ringer’s solution	−1.18 (NEK)	40	0.85	[28]
Mg_63_Zn_32_Ca_5_ (after 15 min immersion)	Ringer’s solution	−1.27 (NEK)	24.7	0.51	[28]
Mg_64_Zn_30_Ca_6_ (after 15 min immersion)	Ringer’s solution	−1.20 (NEK)	33	0.73	[28]
Mg_64_Zn_32_Ca_4_ (after 15 min immersion)	Ringer’s solution	−1.32 (NEK)	24.6	0.51	[28]
Mg_65_Zn_30_Ca_5_ (after 15 min immersion)	Ringer’s solution	−1.21 (NEK)	28	0.63	[28]
Mg_65_Zn_32_Ca_3_ (after 15 min immersion)	Ringer’s solution	–1.32 (NEK)	21	0.43	[28]
Mg_66_Zn_28_Ca_6_ (after 15 min immersion)	Ringer’s solution	–1.21 (NEK)	76	1.67	[28]
Mg_66_Zn_30_Ca_4_ (after 15 min immersion)	Ringer’s solution	–1.34 (NEK)	29	0.64	[28]
Mg_67_Zn_28_Ca_5_ (after 15 min immersion)	Ringer’s solution	−1.26 (NEK)	55	1.17	[28]
Mg_67_Zn_30_Ca_3_ (after 15 min immersion)	Ringer’s solution	−1.26 (NEK)	30	0.64	[28]
Mg_68_Zn_28_Ca_4_ (after 15 min immersion)	Ringer’s solution	−1.35 (NEK)	41	0.88	[28]
Mg_69_Zn_28_Ca_3_ (after 15 min immersion)	Ringer’s solution	−1.27 (NEK)	62	1.33	[28]
Mg	SBF	−1.636	10^−3.96^ (A/cm^2^)	—	[59]
Mg_60_Zn_34_Ca_6_	SBF	—	—	0.06	[55]
Mg_60_Zn_35_Ca_5_ (completely crystalline)	SBF	−1.360	222	331.8 (mpy)	[104]
Mg_60_Zn_35_Ca_5_ (partially amorphous)	SBF	−1.240	4.1	0.1554	[104]
Mg_65.2_Zn_28.8_Ca_6_	SBF	–1.345 ± 0.031	7.50 ± 0.45	—	[76]
Mg_66_Zn_30_Ca_4_	SBF	—	3.53	—	[82]
Mg_66_Zn_30_Ca_4_ (completely crystalline)	SBF	–1.510	1530	2286 (mpy)	[104]
Mg_66_Zn_30_Ca_4_ (partially amorphous)	SBF	–1.270	8.490	12.69 (mpy)	[104]
Mg_67_Zn_29_Ca_4_	SBF	−1.18 (Ref)	18.9	0.21	[105]
Mg_69_Zn_27_Ca_4_	SBF	−1.12	10^−5.81^ A/cm^2^	—	[103]
Mg_70_Zn_25_Ca_5_	SBF	—	11.2	—	[82]
Mg_73_Zn_23_Ca_4_	SBF	—	—	0.21	[55]
Mg_68.5_Zn_27_Ca_4_Mn_0.5_	SBF	−1.235	—	—	[68]
Mg_68_Zn_27_Ca_4_Mn_1_	SBF	−1.254	—	—	[68]
Mg_68_Zn_27_Ca_4_Y_1_	SBF	−1.246	10^−4.96^ (A/cm^2^)	—	[59]
Mg_67_Zn_27_Ca_4_Y_2_	SBF	−1.283	10^−4.78^ (A/cm^2^)	—	[59]
MAO-treated Mg_65.2_Zn_28.8_Ca_6_	SBF	–1.244 ± 0.016	(7.23 ± 0.13) × 10^−2^	—	[76]

E_corr_: corrosion potential, SCE: saturated calomel electrode, i_corr_: corrosion current density, SHE: standard hydrogen electrode, ^1^ Ag/AgCl reference electrode.

## Data Availability

Not applicable.
